# Immature platelet and reticulocyte fractions as dynamic biomarkers of bone marrow activation in dogs and humans: a comprehensive review

**DOI:** 10.1016/j.vas.2026.100786

**Published:** 2026-07-27

**Authors:** Mahmood Ahmadi-hamedani, Kimia Satari

**Affiliations:** a Department of Clinical Sciences, Faculty of Veterinary Medicine, Semnan University, Semnan, Iran; b Student Research Committee, Faculty of Veterinary Medicine, Semnan University, Semnan, Iran

**Keywords:** Immature platelet fraction (IPF), Immature reticulocyte fraction (IRF), Bone marrow activity, Human, Dog

## Abstract

**Background:**

**:** The immature platelet fraction (IPF) is a platelet-related RNA biomarker reflecting thrombopoiesis in vivo and providing immediate information about bone marrow reactivity. Despite being recognized as a reliable marker of erythropoiesis, Immature Reticulocyte Fraction (IRF) is widely utilized whereas IPF is still poorly known in spite of the diagnostic and prognostic value of this marker.

**Objectives:**

**:** To review comprehensively the biology, methodology, and clinical application of IPF in humans and dogs and to assess the complementarity of IPF to IRF.

**Methods:**

**:** The current literature review was performed using PubMed/MEDLINE, Scopus, Web of Science, ScienceDirect, Google Scholar, and Cochrane Library databases. The scientific publications about the biological background, analytical validation, reference values, and clinical usage of IPF and IRF in humans and dogs that have been published until December 2025 were included and analyzed.

**Results:**

**:** Reference intervals of IPF depend on the analyzer used. In normal adults, IPF values are estimated as approximately 1.0–7.3% dependent on the type of Sysmex analyzer. In dogs, IPF obtained using Sysmex XN-1000 V analyzer has a reference interval of 0.5–8%, and IPF lower than 6.9% indicates central thrombocytopenia. In contrast, IRF determined using ADVIA 2120/2120i analyzer in normal large breed dogs has a reference interval of 10.4–43.5% and is significantly increased in regenerative anemia (46.5% vs. 22.1% in controls; AUC = 0.897).

**Conclusion:**

**:** IPF is a dynamic and sensitive marker of thrombopoiesis in human and veterinary medicine. Together with IRF, it allows obtaining more complete information about bone marrow reactivity.

## 1 Introduction

Bone marrow activity is the relationship between the production of hematopoietic elements and the consumption or destruction of blood cells in the periphery. Traditional CBC parameters, platelet count, hemoglobin, and total reticulocyte count yield delayed or indirect evidence of marrow recovery and often fail to detect early regenerative responses (Steinberg & Olver, 2005).Over the past decade, fluorescent based hematology platforms have introduced new parameters of immature circulating cells as real time surrogates of marrow function.

The immature platelet fraction (IPF) and the immature reticulocyte fraction (IRF) indicate the percentage of RNA-rich immature cells released from the marrow.

These indices are sensitive markers of megakaryopoiesis and erythroid activity, respectively (Foy et al., 2015). Both have been shown to be particularly useful in differentiating marrow failure from peripheral cytopenias, monitoring recovery after chemotherapy, and characterizing regenerative vs non-regenerative anemia (Foy et al., 2015; Moloney et al., 2023; Strati et al., 2017Within the framework of One Health and comparative medicine, evaluating IPF and IRF across human and canine species may provide valuable translational insights. Species-specific differences in hematopoiesis, thrombopoiesis, and analyzer calibration need to be accounted for; however, there are many similarities between humans and dogs that allow comparison. For this reason, this review intends to highlight the state-of-the-art knowledge about IPF and IRF in terms of biology, analysis, diagnosis, and clinical use, taking into account the differences between the two species.

## 2 Methods

### 2.1 Literature search strategy

A comprehensive literature search was conducted across multiple electronic databases, including PubMed/MEDLINE, Web of Science, Scopus, Google Scholar, ScienceDirect, and the Cochrane Library. The search included studies published up to December 31, 2025, and was designed to identify relevant literature regarding immature platelet fraction (IPF) and immature reticulocyte fraction (IRF) in both human and canine medicine.

The search strategy combined Medical Subject Headings (MeSH) and free-text keywords, including: (“immature platelet fraction” OR “IPF” OR “reticulated platelets”) AND (“immature reticulocyte fraction” OR “IRF” OR “reticulocyte indices” OR “reticulocyte hemoglobin content” OR “CHr”) AND (“thrombocytopenia” OR “anemia” OR “regenerative anemia” OR “platelet kinetics” OR “bone marrow response”) AND (“dog” OR “canine” OR “human” OR “comparative hematology” OR “clinical pathology”). Additional studies were identified through manual screening of reference lists from relevant review articles and original research papers. The process of searching the literature and selecting the studies was done independently by both authors. Initially, 120 citations were retrieved from the searches in the databases and through the hand searching of references. After eliminating duplicates, 100 citations were left for the next stage of the review. The titles and abstracts were screened for relevancy, and then the studies that were deemed potentially relevant were critically appraised using the inclusion and exclusion criteria that were defined earlier. All disputes about study inclusion were resolved through discussion and consensus. Finally, 21 studies were found to be relevant to the aims of the current review.

### 2.2 Selection criteria

The literature selection process was performed independently by both authors, and any disagreements regarding study eligibility were resolved through discussion and consensus.

The following inclusion criteria needed to be fulfilled by studies considered for the review:

1. Evaluated the biological background, analytical validation, diagnostic utility, prognostic value, and applications of IPF and/or IRF;
2. Aimed to study human and dog patients;
3. Provided the quantitative data on IPF and IRF, such as reference ranges, cut-offs, sensitivity, specificity, predictive power, or data about instrument validation;
4. Carried out using automated hematology analyzers capable of measuring IPF and IRF (Sysmex XN/XE series, Sysmex XN-1000 V, Siemens ADVIA 2120/2120i, Beckman Coulter DxH family);
5. Consisted of peer-reviewed original research, observational, case-control, cohort studies, or analytical validation papers.

The exclusion criteria included the following characteristics:

1. The study subjects other than dogs or humans;
2. The absence of original quantitative data about IPF or IRF;
3. Abstracts of conferences, editorials, letters, expert opinions, non-peer-reviewed articles;
4. Lack of adequate methodology or outcomes data;
5. Duplicate datasets in another publication.

In order to increase the reliability of the review, preference was given to studies that had clear methods, sufficiently large sample size, analyzer-specific reference intervals, and clinically meaningful outcomes. The review articles were mainly utilized for the purpose of obtaining background information and citation search, while numerical data from tables were mainly taken from original studies. Since this particular study is a narrative review and not a systematic or meta-review, no formal risk of bias assessment was done.

### 2.3 Data synthesis

Data were synthesized narratively to evaluate the biological significance, analytical characteristics, and clinical applications of IPF and IRF in human and canine medicine. A formal meta-analysis was not performed because of substantial heterogeneity among studies regarding species, disease categories, analyzer platforms, reported outcome measures, and reference interval methodologies. Consequently, quantitative pooling of results was considered inappropriate and potentially misleading. Instead, findings were compared qualitatively to identify consistent diagnostic trends, analyzer-specific considerations, and translational similarities and differences between humans and dogs. As this work was designed as a narrative review rather than a systematic review, a formal risk-of-bias assessment was not undertaken. Nevertheless, preference was given to peer-reviewed studies with clearly described methodologies, adequate sample sizes, and clinically relevant outcome measures.

## 3 Biological basis of IPF and IRF

### 3.1 Immature platelet fraction (IPF)

Platelets originate from cytoplasmic fragmentation of megakaryocytes and initially retain substantial amounts of residual RNA and metabolic activity. These RNA-rich platelets exhibit increased fluorescence following staining with nucleic acid dyes, allowing fluorescence-based hematology analyzers, particularly the Sysmex XN series, to quantify the immature platelet fraction (IPF) as an indicator of active thrombopoiesis (Buttarello et al., 2020; Foy et al., 2015).

IPF reflects real-time megakaryocytic activity and often changes before alterations in platelet count become apparent.

Increased IPF values are typically associated with enhanced platelet production secondary to peripheral destruction or consumption, whereas decreased values suggest impaired marrow production. The principal interpretive patterns of IPF are summarized in Table 1**.**

**Table 1:** Interpretive patterns of IPF (%) and A-IPF (absolute immature platelet count).

Parameter	Pattern	Interpretation
IPF (%)	↑	Peripheral destruction
IPF (%)	Normal	Reduced marrow response
IPF (%)	↓	Marrow failure
A-IPF (× 10³/µL)	↑	Increased absolute thrombopoiesis
A-IPF (× 10³/µL)	↓	Reduced platelet production

IPF is reported in either percentage form, denoted IPF%, or absolute immature platelet count, A-IPF. The IPF% indicates the ratio of immature platelets to circulating platelet pool, while the A-IPF is the actual count of immature platelets found in peripheral blood. While IPF% is the common reporting value, A-IPF could be used as an added measure for those with severe thrombocytopenia, where IPF% value would be affected by total platelet count (Buttarello et al., 2020; Hoffmann, 2014).

Because IPF measurements are influenced by analytical methodology, fluorescence thresholds, and analyzer-specific calibration algorithms, reference intervals are platform dependent and should not be interpreted interchangeably across different hematology systems. Consequently, both species-specific and analyzer-specific considerations are required when interpreting IPF results. Representative reference intervals reported in healthy human and canine populations are summarized in Table 2**.**

**Table 2:** Published reference data for immature platelet fraction (IPF) in healthy human and canine populations.

Species	Population	Age/Sex information	Analyzer	N	IPF (%)	Reference Interval (%)	Reference
Human	Healthy adults	Both sexes; no clinically relevant sex partition required	Sysmex XE-2100	300	3.4 ± 1.8	1.1–6.1	Abe et al.2006
Human	Healthy adults	Both sexes	Sysmex XN series	247	2.9 ± 1.7	1.3–8.0	Abe et al.2006
Human	Healthy adults	Both sexes	Sysmex XN-10	236	3.3 ± 1.8	1.6–7.1	Abe et al.2006
Dog	Healthy dogs	Mixed breeds; demographic characteristics reported in original study	Sysmex XN-1000V	803	Median 3.0 (IQR 2.9)	0.5–8.0	Pérez-Ecija et al., 2024

Abbreviations: IPF, immature platelet fraction; IQR, interquartile range.

Note: Reference intervals were gathered from the literature and might vary based on the population studied, analyzing system used, and methodology applied. Most reference ranges available for veterinary IPF analysis are based on Sysmex fluorescence analyzing systems.

The reference intervals for IPF must be cautiously interpreted as there are several factors that affect them, such as species, age, method of analysis, fluorescence cut-off levels, and algorithm of the instrument itself. While there were significant differences in results based on gender in some human studies, these differences were regarded as small enough to establish separate reference intervals based on the sex of the patient. In addition, most of the literature available regarding reference intervals for veterinary IPF analysis comes from Sysmex fluorescence analyzing systems, specifically the Sysmex XN-1000 V one. Therefore, direct numerical comparison of IPF results between the systems is discouraged, and analyzer-specific reference intervals based on the species/population are preferred whenever possible (Buttarello et al., 2020).

### 3.2 Immature reticulocyte fraction (IRF)

Reticulocyte maturation represents the final stage of erythropoiesis before mature erythrocytes are released into circulation. Based on residual intracellular RNA content, reticulocytes can be subdivided into low-fluorescence (LFR), medium-fluorescence (MFR), and high-fluorescence (HFR) populations. The immature reticulocyte fraction (IRF) corresponds to the proportion of MFR and HFR cells and therefore reflects the earliest stages of circulating reticulocyte maturation (Ahmadi-Hamedani & Bagherian, 2025; Jung et al., 2023).

IRF is believed to be an early marker for erythropoiesis due to its rise prior to any alteration in hemoglobin level or reticulocyte count. In case of regenerative anemia, blood loss, hemolysis, or recovery from bone marrow suppression, IRF can show its increase prior to hematological markers exhibiting regeneration (Jung et al., 2023; Moloney et al., 2023; Strati et al., 2017).

Interpretation of IRF should always be performed within the clinical context. Although increased IRF generally reflects enhanced erythropoietic activity, normal IRF values do not necessarily exclude regenerative responses. Disease duration, timing of sample collection, species-specific erythropoietic kinetics, erythropoietin stimulation, and pre-analytical variables may all influence IRF results. Consequently, IRF should be interpreted together with reticulocyte counts, hematologic findings, and clinical presentation rather than as a standalone marker (Jung et al., 2023; Piva et al., 2015).

From a methodological perspective, IRF measurements are analyzer-dependent. ADVIA analyzers classify reticulocyte maturity using optical light-scatter cytochemistry, whereas Sysmex analyzers employ fluorescence flow cytometry based on residual RNA content. Sysmex fluorescence-based methods are generally considered to provide greater sensitivity for detecting subtle changes in reticulocyte RNA content, whereas ADVIA analyzers provide well-established erythrocyte and reticulocyte indices that have been extensively validated in both human and veterinary hematology. However, both platforms have demonstrated satisfactory analytical performance and clinical utility when interpreted using analyzer-specific reference intervals (Buttarello et al., 2020; Piva et al., 2015). Importantly, classification of reticulocyte maturity fractions is not fully standardized among manufacturers. Differences in staining procedures, gating strategies, and maturity thresholds may result in numerical discrepancies between analyzers. Therefore, IRF values obtained from different analytical systems should not be interpreted interchangeably, and studies using different platforms should be compared primarily in terms of biological trends and clinical relevance rather than direct numerical equivalence (Buttarello et al., 2020).

The principal clinical applications and interpretive patterns of IRF are summarized in Table 3, whereas representative analyzer-specific reference intervals reported for healthy humans and dogs are presented in Table 4.

**Table 3:** Clinical interpretation of IRF (%) in different clinical settings.

Clinical condition	IRF (%) pattern	Clinical interpretation	References
Regenerative anemia	↑ Increased	Early marrow response with release of immature reticulocytes	Jung et al., 2023; Strati et al., 2017
Hemolytic anemia	↑ Increased	Accelerated erythropoiesis secondary to RBC destruction	Piva et al., 2015
Acute blood loss	↑ Increased	Compensatory erythroid regeneration	Piva et al., 2015
Recovery after chemotherapy / marrow suppression	↑ Increased	Early indicator of marrow recovery	Moloney et al., 2023; Strati et al., 2017
Non-regenerative anemia	Normal or mildly increased	Reduced erythropoietic responsiveness	Jung et al., 2023
Severe marrow failure / aplastic anemia	↓ Decreased	Markedly reduced erythroid production	Piva et al., 2015

Note1: All values refer to percentage-based immature reticulocyte fraction measurements (IRF%). Human reference intervals were derived from a Sysmex XE-5000 fluorescence-based platform, whereas canine reference intervals were primarily generated using ADVIA 2120/2120i analyzers. Because IRF measurements depend on analyzer methodology, staining procedures, and gating algorithms, direct numerical comparison between platforms should be interpreted with caution and analyzer-specific reference intervals should be applied.

**Table 4:** Published IRF (%) reference data in healthy humans and dogs.

Species	Population characteristics	Analyzer	N	IRF (%)	Reference interval (%)	Reference
Human	Healthy adults; mean age 44 y (13–80 y); 54.5% female	Sysmex XE-5000	132	Median 5.35	1.6–12.1	Morkis et al., 2016
Dog	Healthy large-breed adult dogs; mixed sex	ADVIA 2120	106	NR	10.4–43.5	Moloney et al., 2023
Dog	Healthy dogs included as controls in a clinical study	ADVIA 2120i	45	22.7 ± 9.4	Not reported	Jung et al., 2023

Note2: The canine data reported by Jung et al. (2023) represent healthy control animals included in a clinical study and should not be interpreted as an independently established reference interval.

## 4 Clinical and translational significance of IPF

### 4.1 Diagnostic significance

In acute immune thrombocytopenia (ITP), IPF is markedly increased despite low platelet counts, distinguishing peripheral destruction from bone marrow failure. Conversely, concurrent decreases in IPF and platelet count indicate hypocellular or therapy-induced marrow suppression. In dogs, similar patterns are reproducible and often parallel reticulated platelet counts measured by flow cytometry (Abe et al., 2006, 2008).

### 4.2 Prognostic and monitoring relevance

Serial measurements of IPF allow dynamic monitoring of marrow recovery after chemotherapy, infection, or myelosuppressive therapy. Rising IPF values typically precede platelet count recovery by 1–3 days, offering an early indicator of regenerative response (Ahmadi-Hamedani et al., 2025; Hoffmann, 2014).

### 4.3 Translational applicability

Because platelet production kinetics and RNA degradation rates are comparable across mammals, IPF exhibits translational potential from human medicine to veterinary hematology. The marker provides a model for studying regenerative kinetics, treatment response, and comparative thrombopoietic physiology (Ahmadi-Hamedani et al., 2025; Briggs, 2009).

## 5 Analytical platforms and measurement principles

The measurement of IPF and IRF is tightly linked to the analytical platform employed. Each hematology system uses distinct fluorescence dyes, cell-gating algorithms, and species-specific calibration settings, resulting in platform-dependent numerical values and reporting styles. Consequently, reference intervals and clinical cut-offs cannot be applied interchangeably across instruments. (Table 5)

**Table 5:** Comparison of major hematology platforms used for IPF and IRF measurement.

Platform	Primary parameters	Human use	Canine use	References
Sysmex XE/XN	IPF (%), A-IPF (× 10³/µL)	✓	✓	Abe et al., 2006; Buttarello et al., 2020
Sysmex XN-V	IPF (%), A-IPF (× 10³/µL)	—	✓	Pérez-Ecija et al., 2024
ADVIA 2120/2120i	IRF (%)	✓	✓	Moloney et al., 2023
Beckman Coulter DxH 800	IRF (%)	✓	—	Piva et al., 2015
Mindray BC-6800	IPF (%)	✓	Limited	Buttarello et al., 2020

Because most veterinary IPF data originate from Sysmex XN-V/XN-1000V analyzers and the majority of veterinary IRF studies rely on ADVIA 2120/2120i**,** the available evidence is inherently instrument-weighted. Human studies similarly cluster by platform: IRF is more frequently reported on ADVIA systems, whereas IPF is primarily described using Sysmex fluorescence channels.

Although dogs and humans might show analogous physiological responses with respect to bone marrow regeneration, an arbitrary species difference should not be translated to absolute reference ranges, because reference ranges are affected by species-specific dynamics of erythropoiesis and thrombopoiesis and analyzer specific measurement principles. Therefore, cross-species comparisons should focus on biological and diagnostic relevance rather than direct numerical equivalence of reference intervals or cut-off values. This analyzer dependency highlights the need for standardized multicenter validation studies and platform-specific reference interval verification before broader clinical implementation of IPF and IRF in veterinary medicine (Ahmadi-Hamedani et al., 2025; Buttarello et al., 2020)

## 6 IPF and IRF in regenerative vs non-regenerative cytopenias

Bone marrow response to cytopenias depends on the lineage involved, the underlying mechanism (destruction vs. decreased production), and timing of sampling. IPF and IRF provide lineage-specific and time-sensitive indicators of hematopoietic activation (Abe et al., 2006, 2008; Briggs, 2009) (Table 6).

**Table 6:** Conceptual interpretation of IPF and IRF responses across canine and human studies.

Condition	IRF (%) pattern	IPF (%) pattern	Interpretation / marrow activity
Regenerative anemia	↑↑ (HFR + MFR expansion)	Normal / ↑	Active erythropoiesis; effective reticulocyte release
Non‑regenerative anemia	↓ / Normal	Normal / ↓	Ineffective or reduced marrow output
Immune thrombocytopenia (ITP)	Normal	↑↑ (> 10–15 %)	Isolated platelet regeneration; peripheral platelet loss
Bone marrow suppression	↓	↓	Global marrow inactivity or aplasia

Note: All IPF and IRF values shown in this table refer to percentage-based measurements (IPF% and IRF%). The table summarizes general interpretive patterns derived from both human and canine studies; exact reference intervals and diagnostic cut-off values remain analyzer- and species-specific.

### 6.1 Regenerative anemia

High Fluorescence Ratio (HFR) and Medium Fluorescence Ratio (MFR) represent the most immature reticulocyte populations, collectively forming the Immature Reticulocyte Fraction (IRF). Their simultaneous elevation is a hallmark of an aggressive erythropoietic rebound, reflecting a rapid marrow response (Moloney et al., 2023; Strati et al., 2017).

• **IRF:** markedly elevated (↑ HFR + ↑ MFR) — earliest sign of erythropoietic rebound.
• **IPF:** normal or mildly increased unless concurrent thrombopoietic drive exists.
• **Kinetics**: In many regenerative conditions, IRF may increase earlier than the absolute reticulocyte count; however, the timing of this response varies according to species, disease severity, erythropoietic stimulus, and sampling interval (Jung et al., 2023; Piva et al., 2015).

### 6.2 Non-regenerative anemia

Occurs with aplastic anemia, myelofibrosis, Chronic kidney disease (CKD), or inflammatory disease (Abe et al., 2006; Foy et al., 2015)

• **IRF:** low or normal → impaired erythropoietic output.
• **IPF:** normal unless megakaryopoiesis is also suppressed.

### 6.3 Immune thrombocytopenia (ITP)

• **IPF:** Is often elevated in cases of immune-related thrombocytopenia, but the cut-offs reported vary across species, analyzer used, age of the individual, reference population, and study methodology. Therefore, a universal cutoff cannot be recommended, and analyzer-based reference ranges are needed (Briggs et al., 2004; Buttarello et al., 2020; Pérez-Ecija et al., 2024)
• **IRF:** remains within normal range → isolated megakaryocytic activation.

### 6.4 Bone marrow suppression

Following chemotherapy, radiation, or extensive infiltration. (Pérez-Ecija et al., 2024)

• **IRF:** low
• **IPF:** low

## 7 Diagnostic performance and cut-off evaluation of IPF

Several clinical and experimental studies across human and veterinary medicine demonstrate that immature cell indices outperform conventional CBC parameters in detecting early marrow response. While IRF has historically been emphasized as a sensitive marker of erythroid regeneration, accumulating evidence indicates that IPF provides equal or superior diagnostic value, particularly in thrombocytopenic states and treatment-response monitoring. (Schaefer & Stokol, 2015) (Table 7).

**Table 7:** Diagnostic performance and reported cut-off or reference values of IPF and IRF across different species and analytical platforms.

Species	Biomarker	Analyzer	Reported cut-off / Reference value	Clinical application	Diagnostic implication	Reference
Human	IPF (%)	Sysmex XE-5000	Reference interval: 0.8–5.6% (median 2.4%)	Healthy adult reference population	Baseline values for interpretation of thrombopoietic activity	Morkis et al., 2016
Human	IRF (%)	Sysmex XE-5000	Reference interval: 1.6–12.1% (median 5.35%)	Healthy adult reference population	Baseline values for assessment of erythropoietic activity	Morkis et al., 2016
Dog	IPF (%)	Sysmex XN-1000V	6.3% (optimal ROC cut-off)	Differentiation of peripheral vs. central thrombocytopenia	Higher values favor peripheral platelet destruction	Pérez-Ecija et al., 2024
Dog	Absolute IPF (IPFc) (× 10³/µL)	Sysmex XN-1000V	7.3 × 10³/µL (optimal ROC cut-off)	Differentiation of peripheral vs. central thrombocytopenia	Improves discrimination when interpreted with platelet count	Pérez-Ecija et al., 2024

Note: The diagnostic cut-offs reported in this table have been established based on Sysmex fluorescence hematological analyzers. Since IPF and IRF results depend on the instrument used, the above-mentioned cut-offs cannot be used for the analyzers of other brands without proper validation. Currently, there are cut-offs validated for diagnosis of IPF and IRF only for Sysmex-based analyzers.

### 7.1 Under-recognized diagnostic value of IPF

IPF has received comparatively less attention despite reflecting a more dynamic and rapidly responsive lineage. Platelet turnover is faster than erythrocyte production, allowing IPF to rise earlier and with greater amplitude following marrow stimulation. This kinetic advantage makes IPF particularly valuable for:

• Early prediction of platelet recovery after chemotherapy
• Differentiation of peripheral destruction (e.g., ITP) from marrow failure
• Monitoring short-term therapeutic response in critical or hospitalized patients

### 7.2 Diagnostic cut-offs and predictive performance

Defined IPF and IRF thresholds have demonstrated clinically meaningful predictive power:

• IPF > 7 % in chemotherapy patients predicts platelet recovery within 48 h
• IRF > 15 % anticipates erythroid recovery prior to hemoglobin increase
• In dogs, IRF > 35 % differentiates regenerative from non-regenerative anemia with > 85 % sensitivity

Reported diagnostic cut-off values for IPF vary substantially according to species, analyzer platform, analytical methodology, study population, and clinical context. Consequently, no universal IPF threshold can be recommended, and published cut-offs should always be interpreted within analyzer-specific reference intervals.

## 8 Integrated clinical interpretation of IPF and IRF

### 8.1 Why is integrated interpretation important?

The current section concentrates on the clinical interpretation of IPF and IRF in combination rather than their correlation, as the evidence is derived from heterogeneous studies performed with different species, analytical platforms, and designs, making it impossible to perform any kind of correlation analysis in the scope of this narrative review.

While IPF and IRF characterize the bone marrow activity, they indicate two distinct hematopoietic lineages. The former parameter characterizes the megakaryopoiesis activity, while the latter one indicates the erythropoietic regeneration. The interpretation of both parameters in tandem provides additional information on the responsiveness of specific hematopoietic lineages and increases the possibilities of differential diagnosis of peripheral cell destruction versus bone marrow production impairment (Briggs, 2009; Buttarello et al., 2020).

An increase in both IPF and IRF suggests simultaneous activation of thrombopoiesis and erythropoiesis, which can be seen in the bone marrow recovery after chemotherapy, stem-cell transplantation, or hemorrhage.

On the other hand, IRF elevation and IPF normalization suggest selective erythroid regeneration, for example, in regenerative anemia, hemolytic anemia, or acute blood loss.

In contrast, an elevated IPF with a normal IRF is indicative of stimulation of the thrombopoiesis process due to peripheral destruction of platelets, including immune thrombocytopenia (ITP), or increased consumption of platelets (Jung et al., 2023; Pérez-Ecija et al., 2024; Piva et al., 2015).

Decrease of both of these indicators simultaneously can indicate general bone marrow suppression or failure, which can include aplastic anemia, bone marrow infiltration, late stages of myelosuppression after chemotherapy, or chronic disorders of bone marrow. Since both the parameters depend upon the analysis technique, animal-specific kinetics of the hemopoiesis process, disease stage, and pre-analytical factors, their interpretation should always take into account the results of the complete blood count analysis, along with the platelets and reticulocytes absolute values (Buttarello et al., 2020; Briggs, 2009).

The interpretive patterns summarized in Table 8 illustrate the complementary diagnostic value of simultaneous IPF (%) and IRF (%) assessment rather than a direct statistical correlation between the two biomarkers. Because platelet and erythrocyte production are regulated through distinct hematopoietic lineages, discordant changes in IPF and IRF may provide important information regarding lineage-specific bone marrow responses. For example, isolated elevation of IPF is more consistent with increased thrombopoiesis secondary to peripheral platelet destruction, whereas isolated elevation of IRF reflects selective activation of erythropoiesis during regenerative anemia or recovery following blood loss. Concurrent elevation of both parameters may indicate generalized marrow regeneration after myelosuppressive injury or recovery from bone marrow suppression, while simultaneous reduction of both indices suggests impaired multilineage hematopoiesis. Because both IPF and IRF are influenced by species, analytical platform, and instrument-specific reference intervals, these patterns should be interpreted according to analyzer-specific reference ranges and clinical context rather than universal numerical cut-off values (Buttarello et al., 2020; Piva et al., 2015; Pérez-Ecija et al., 2024).

**Table 8:** Integrated clinical interpretation of percentage-based immature platelet fraction (IPF%) and immature reticulocyte fraction (IRF%) patterns in dogs and humans.

IPF (%)	IRF (%)	Biological interpretation	Representative clinical conditions	Evidence	Key references
↑	↑	Simultaneous activation of megakaryopoiesis and erythropoiesis	Bone marrow recovery after chemotherapy, stem-cell transplantation, severe hemorrhage	Human studies	Buttarello et al., 2020; Strati et al., 2017
Normal	↑	Predominantly erythroid regeneration	Regenerative anemia, hemolytic anemia, acute blood loss	Human and canine studies	Jung et al., 2023; Moloney et al., 2023; Piva et al., 2015
↑	Normal	Predominantly megakaryocytic regeneration	Immune thrombocytopenia, peripheral platelet destruction	Human and canine studies	Briggs et al., 2004; Pérez-Ecija et al., 2024
↓	↓	Global bone marrow hypoproliferation	Aplastic anemia, chemotherapy-induced marrow suppression, marrow infiltration	Human studies	Briggs, 2009; Buttarello et al., 2020

## 9 Discussion

Both immature platelet fraction (IPF) and immature reticulocyte fraction (IRF) have proved to be dynamic hematologic biomarkers giving the earliest possible information about the bone marrow activity as compared to classical platelet and reticulocyte counts. Since immature circulating cells become apparent soon after thrombopoietic or erythropoietic stimulation, such parameters represent the current activity of bone marrow and may reveal the process of regeneration prior to any changes in mature cell population (Briggs, 2009; Buttarello et al., 2020; Piva et al., 2015).

According to the existing data, IPF is a very helpful parameter to assess thrombocytopenia. Elevated IPF means that there is preserved or increased megakaryopoiesis due to destruction/consumption of platelets, while low/normal IPF in patients with thrombocytopenia implies poor platelet production. Similarly, IRF is used to evaluate early erythroid regeneration and is elevated in the course of the recovery of regenerative anemia, acute bleeding or bone marrow suppression before absolute reticulocyte counts (Jung et al., 2023; Moloney et al., 2023; Strati et al., 2017).

Despite the high degree of biological similarity between humans and dogs regarding the biology of thrombopoiesis and erythropoiesis, several important species-specific distinctions must be mentioned. Hematopoiesis in canines differs from that in humans in terms of platelet dynamics, erythroid dynamics, and regeneration capacity. As a result, reference intervals and diagnostic cut-offs differ. Thus, direct comparison of IPF and IRF values between species is not appropriate. Comparative analysis should be based on similarities in biological behavior and diagnostic characteristics instead of absolute agreement in numbers (Moloney et al., 2023; Pérez-Ecija et al., 2024).

One of the key aspects revealed in the current review is the significant impact of the analytical technique on the measurement of immature cells. The IPF was mainly assessed via Sysmex analyzers, while the majority of canine IRF reference data were obtained via ADVIA 2120/2120i. As a result, since each analyzer uses specific methods of staining, fluorescence detection, gating, and analysis algorithm, their results cannot be interpreted interchangeably. Therefore, the importance of analyzer-specific reference intervals remains significant (Buttarello et al., 2020; Piva et al., 2015).

However, there is a range of drawbacks associated with these biomarkers. Firstly, veterinary studies exploring IPF and IRF lack sufficient data compared to human research. Secondly, the diversity of methods applied within different studies is high in regard to such aspects as analyzer systems, patients' groups, criteria of diseases definition, and reporting practices. Thirdly, a great number of studies provide information about percentage indices of immature cells, but do not offer corresponding absolute immature cell counts. Fourthly, standard analyzer-specific reference intervals are still lacking for a number of veterinary systems, in particular, for IRF calculated via fluorescence analyzers.

Prospective multicenter studies with consistent methodology and well-defined research population should be conducted in future in order to provide an improved basis for comparative analysis. The establishment of reference intervals, percentage-based and absolute indices, as well as harmonization of methods of calculations will contribute to further application of IPF and IRF in the field of hematology.

Generally speaking, the existing data indicate that IPF and IRF should be considered complementary markers and not competing ones. The combination of IPF and IRF reflects the activity of bone marrow lineage better than either of the parameters on its own.

## 10 Conclusion

The immature platelet fraction (IPF) and the immature reticulocyte fraction (IRF) offer clinically relevant data concerning ongoing thrombopoeitic and erythopoeitic activities that add valuable information to the traditional complete blood count variables. Since these immature cell parameters often alter even before changes occur in mature circulating cells, they allow early detection of bone marrow response, differentiation of peripheral destruction versus reduced production, and assessment of hematopoietic recovery. There is already enough scientific evidence demonstrating the relevance of IPF and IRF in both human and veterinary medicine. Nevertheless, the results of any interpretation should take into account species-related physiology, the technique used by a particular analyzer, and reference intervals for this analyzer. Therefore, numerical data and diagnostic cut-off points measured with various hematology analyzers cannot be considered equivalent. Despite an ongoing progress of the veterinary literature, it is necessary to conduct additional multicenter prospective research in order to develop standardized methodologies, reference intervals and cut-offs of analyzers, and evaluate clinical relevance of immature cell parameters.

## Statement for studies in humans and animals

This article is a review article and does not contain any studies with human participants or animals performed by the authors.

## CRediT authorship contribution statement

**Mahmood Ahmadi-hamedani:** Writing – review & editing, Writing – original draft. **Kimia Satari:** Writing – review & editing, Writing – original draft, Methodology.

## Declaration of competing interest

The authors declare that they have no known competing financial interests or personal relationships that could have appeared to influence the work reported in this paper.
